# Nanoparticle administration method in cell culture alters particle-cell interaction

**DOI:** 10.1038/s41598-018-36954-4

**Published:** 2019-01-29

**Authors:** Thomas L. Moore, Dominic A. Urban, Laura Rodriguez-Lorenzo, Ana Milosevic, Federica Crippa, Miguel Spuch-Calvar, Sandor Balog, Barbara Rothen-Rutishauser, Marco Lattuada, Alke Petri-Fink

**Affiliations:** 1 Adolphe Merkle Institute, Université de Fribourg, Fribourg, 1700 Switzerland; 2 Chemistry Department, Université de Fribourg, Fribourg, 1700 Switzerland

## Abstract

As a highly interdisciplinary field, working with nanoparticles in a biomedical context requires a robust understanding of soft matter physics, colloidal behaviors, nano-characterization methods, biology, and bio-nano interactions. When reporting results, it can be easy to overlook simple, seemingly trivial experimental details. In this context, we set out to understand how *in vitro* technique, specifically the way we administer particles in 2D culture, can influence experimental outcomes. Gold nanoparticles coated with poly(vinylpyrrolidone) were added to J774A.1 mouse monocyte/macrophage cultures as either a concentrated bolus, a bolus then mixed via aspiration, or pre-mixed in cell culture media. Particle-cell interaction was monitored via inductively coupled plasma-optical emission spectroscopy and we found that particles administered in a concentrated dose interacted more with cells compared to the pre-mixed administration method. Spectroscopy studies reveal that the initial formation of the protein corona upon introduction to cell culture media may be responsible for the differences in particle-cell interaction. Modeling of particle deposition using the *in vitro* sedimentation, diffusion and dosimetry model helped to clarify what particle phenomena may be occurring at the cellular interface. We found that particle administration method *in vitro* has an effect on particle-cell interactions (i.e. cellular adsorption and uptake). Initial introduction of particles in to complex biological media has a lasting effect on the formation of the protein corona, which in turn mediates particle-cell interaction. It is of note that a minor detail, the way in which we administer particles in cell culture, can have a significant effect on what we observe regarding particle interactions *in vitro*.

## Introduction

The increasing use of engineered nanomaterials in consumer products, development as diagnostic tools and therapeutic vectors, and growing awareness regarding nano-sized pollutants and environmental toxicology has led to a significant push in nanoscience research. Despite the prolific research output, in particular in the fields of nanomedicine and nanotoxicology, there has been mixed success translating biomedical nanoparticles (NPs) from the lab into the clinic^1–5^. The challenges in bridging the gap between bench-top work and clinical success may stem from difficulty in conducting large-scale synthesis of NPs, our limited understanding of fundamental bio-nano interactions, lack of worldwide standardization in the field regarding NP characterization and biological assays, and widespread inter-laboratory differences in techniques and methodology leading to difficulty reproducing work^6–8^. Furthermore, variation in experimental design, protocols, controls, and execution may influence experimental outcomes and hinder inter-laboratory reproducibility.

As a prominent example of the difficulty in standardizing experiments where nanoparticles-cell interactions are addressed, we investigated whether *in vitro* administration, i.e., the literal way in which researchers add NPs to cells, influenced the outcome of NP-cell interaction studies (i.e. NP uptake and adsorption to the cell surface). Particle administration could influence a number of factors such as the local environment around NPs, the distribution of NPs within the experimental media. It is therefore expected that the way in which NPs are administered (e.g. as a homogeneous suspension or as a concentrated bolus) would influence particle-cell interactions. Moreover, this could influence inter-particle interactions, NP colloidal stability, or protein adsorption.

*In vitro*, the ideal case is to expose cells to a colloidally stable, homogenous dispersion of NPs. However, this is not always possible given certain experimental restrictions–for example live-cell imaging setups can be sensitive to minor fluctuations in temperature, which can cause a shift in the imaging focal plane. Therefore, NPs may need to be administered as a concentrated dose that dilutes to the desired experimental concentration. We hypothesized that NP administration *in vitro* would influence the observed NP-cell interactions. There is a large body of work investigating the effect of particle physico-chemical properties (e.g. size, shape, surface charge and functionalization, material, hydrophilicity/hydrophobicity, etc.) on cellular uptake of particles^9–12^, and these factors definitely influence particle-cell interaction. However, issues of reproducibility in science persist, and these may be caused by lack of reporting, non-standardized characterization of nanomaterials, variations in biological assays, etc^13^.

**Figure 1:**
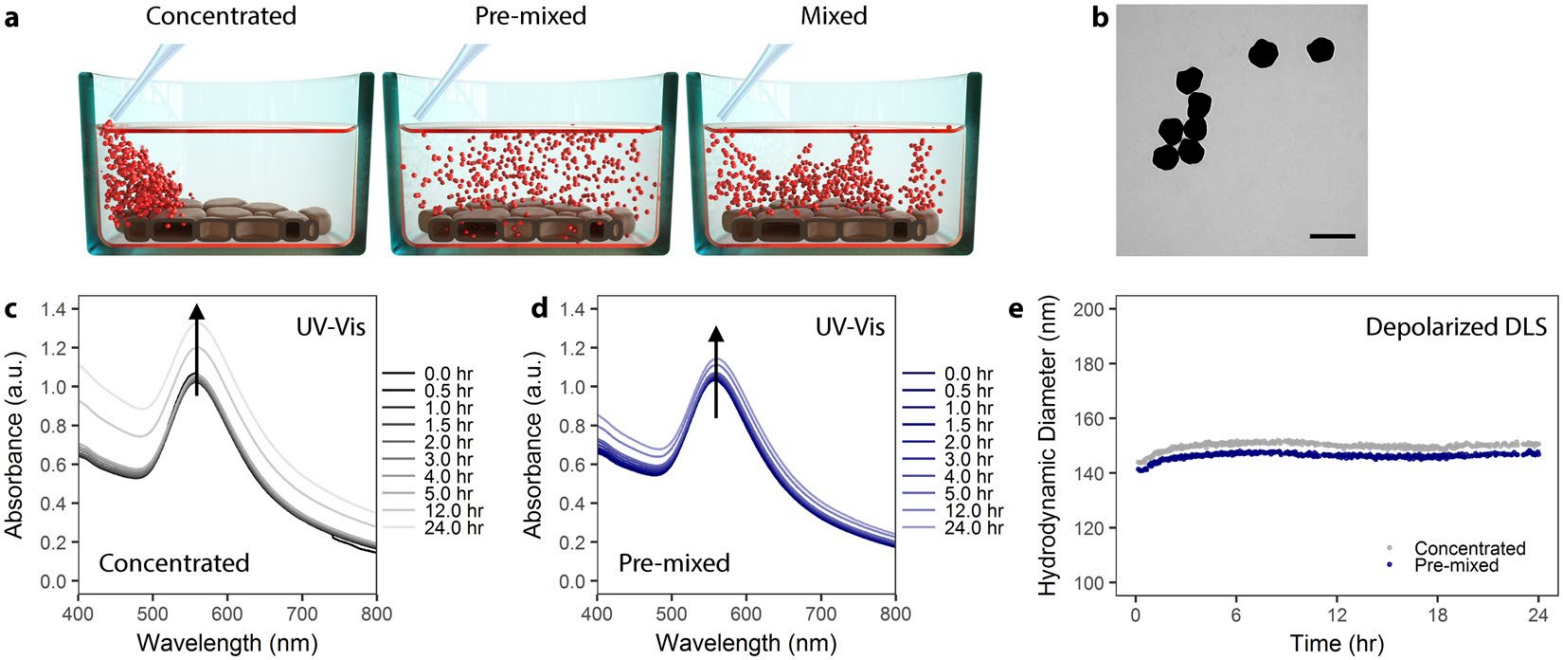
AuNP colloidal stability is independent of administration method. (**a**) Schematic showing the different administration method concepts. Particles can be administered to cells as a concentrated dose (left), a pre-mixed solution (center), or as a concentrated dose which is mixed via aspiration (right). (**b**) Transmission electron microscopy of citrate AuNP with shows a mean core diameter of 116 ± 12 nm (n = 289). Scale bar represents 200 nm. (**c**) UV-Vis spectra of PVP-coated AuNPs over 24 hr in cell culture media supplemented with 10% fetal bovine serum (FBS) following concentrated administration. (**d**) UV-Vis spectra of PVP-coated AuNPs administered via the pre-mixed method in cell culture media supplemented with 10% FBS. (**e**) Depolarized dynamic light scattering of PVP-coated AuNPs measured over 24 hr in complete cell culture media.

The effect of particle administration *in vitro* on particle-cell interaction could be mediated through, for example, an effect on NPs colloidal stability, NP-protein interactions, or changing NPs sedimentation velocity/fluid dynamics^14^. Figure 1a provides a schematic representation of how NPs may be administered via different means. More generally stated, there are widespread inter-laboratory differences in cell culture that, when unrecognized or unacknowledged, may hinder reproducibility and proper interpretation of results. In fact, there have been numerous initiatives to improve reproducibility and confidence in biomedical research^15^ and particle administration may be a variable which has been unacknowledged to date.

In the following work, we show that *in vitro* administration of NP can influence cellular uptake and NP-cell association. Moreover, we emphasize the critical influence of particle surface, and necessity for proper NP characterization. We utilized polyvinylpyrrolidone (PVP)-coated, 116 nm diameter gold nanoparticles (AuNP) as a model particle system to study the effects of *in vitro* administration on particle-cell interaction (Figs 1b and S1). AuNP were used because they are, from an analytical standpoint, simpler to detect. Moreover, due to surface plasmon effects we could qualitatively evaluate the formation of a protein corona around the NPs. We chose PVP as a stabilizing polymer because it is biocompatible, hydrophilic, and used commonly as a NP-stabilizing ligand^16–18^. Here, we favored the use of PVP^19–21^ over a more common polymeric coating such as poly(ethylene glycol) (PEG) due to the tendency of PEG to minimize particle uptake^22,23^. 116 nm AuNP-PVP were administered to cells following three different administration paradigms: (1) NPs pre-mixed in complete cell culture media prior to cell exposure, (2) NPs administered as a concentrated dose to cells in media and then mixed via pipetting, or (3) NPs administered as a concentrated dose to cells in media without mixing (Fig. 1a).

At this particular size, NP deposition and exposure to cells will be driven by particle sedimentation^24^. Thus the transport is simple, i.e. linear in time. Comparatively, transport of small NPs (i.e. <30 nm) is more heavily influenced by diffusion rather than sedimentation, and is non-linear in time^25^. Moreover, for larger NPs polydispersity is moderate and the influence of a non-instantaneous particle association to the cell is negligible, that is small particles may “diffuse away” if not immediately adherent to the cell surface while larger NPs “sit” on the cell. We anticipated that 116 nm AuNPs would emphasize any effects of pipetting on NP-cell interactions. Prior to *in vitro* cellular deposition and uptake studies, NPs were fully characterized in complete cell culture media via UV-Vis spectroscopy and depolarized dynamic light scattering (DDLS). NP dosimetry was simulated following the *in vitro* sedimentation, diffusion, and dosimetry (ISDD) model^26–29^. This model was further developed in order to account for administration-dependent interactions and model the observed phenomena. To fully understand this phenomenon, we also investigated 100 nm PVP-coated SiO_2_ NP, and small, approximately 22 nm PVP-coated iron oxide NPs.

## Results and Discussion

### Nanoparticles are stable in complete cell culture medium

Ensuring colloidal stability of NPs is not a trivial challenge when considering the behavior of NPs in complex biological media (e.g. blood or cell culture media containing serum). Upon introduction into media, macromolecules such as proteins and/or lipids will rapidly adsorb onto the NPs surface^30–33^. This event, coupled with the dynamic environment (i.e. high salt concentration, presence of electrolytes or other molecules, shifting pH), can result in a number of effects on NPs including colloidal destabilization and NP aggregation, competitive adsorption/desorption of macromolecules resulting in the loss of stabilizing ligands on the NP’s surface, and NP dissolution^14,34^. Therefore it is critical to characterize NPs in their relevant environment or media. Neglecting to consider these interactions may lead to difficulty or mistakes when interpreting data, or result in works wholly irreproducible by others^35^.

Colloidal stability of PVP-coated AuNP in 10% fetal bovine serum (FBS) supplemented, complete Dulbecco’s Modified Eagle’s Medium (cDMEM) was studied using UV-Vis spectroscopy and DDLS over 24 hr at 37 °C (Fig. 1c–e). We added NPs to media via the pre-mixed or concentrated approaches in order to study potential NP aggregate formation due to the administration method. Figure 1c,d shows the evolution of the UV-Vis spectra of AuNPs and reveals that the NPs remain colloidally stable, as there is no significant peak broadening when administered via either the concentrated or pre-mixed method^36,37^. This colloidal stability was confirmed by DDLS (Fig. 1e), demonstrating that PVP coating confers colloidal stability to NPs via steric repulsion, even under high salt conditions^38^.

### Nanoparticle administration affects particle interaction with macrophages

**Figure 2:**
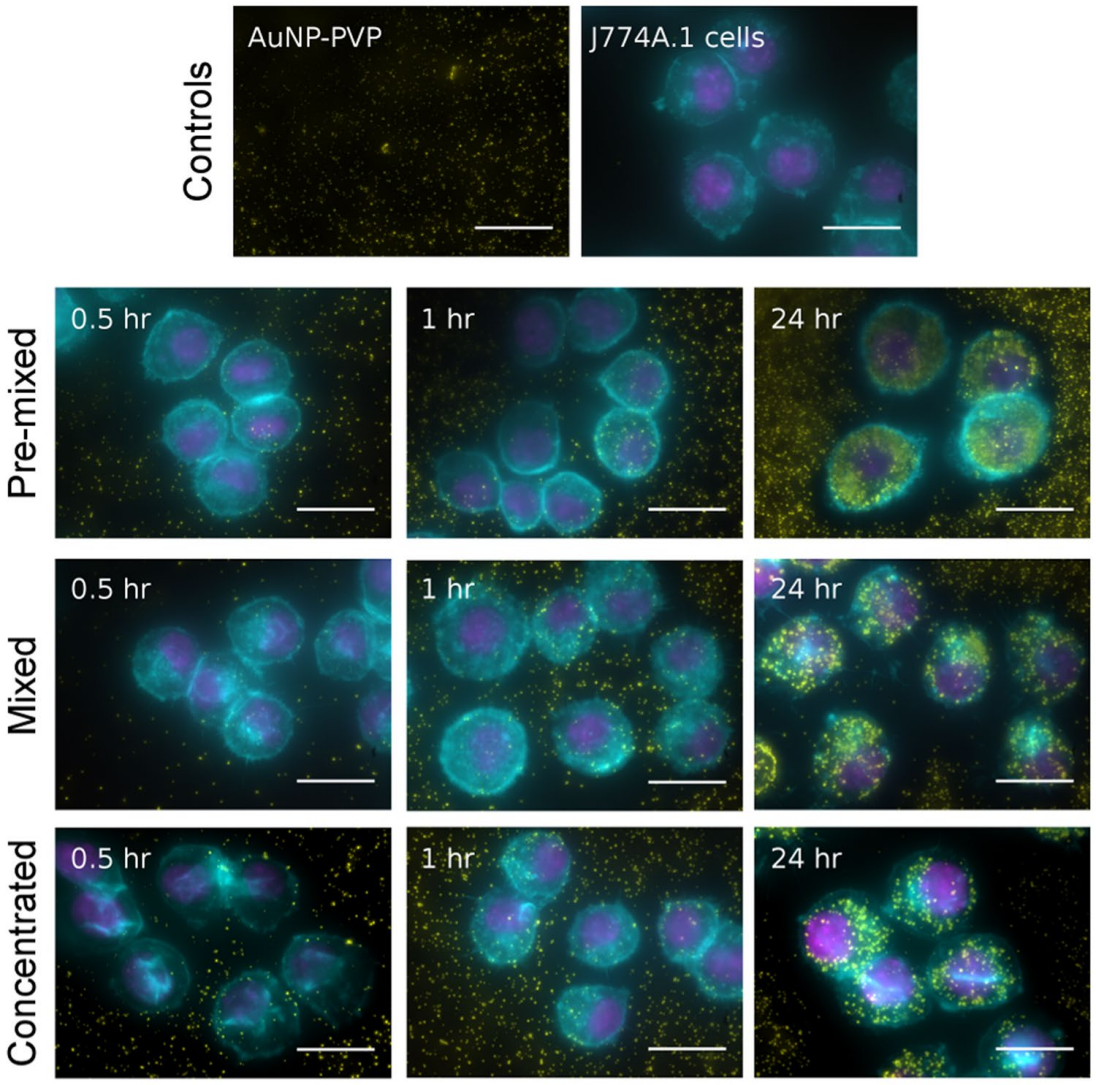
The deposition/uptake of AuNP-PVP (yellow) on J774A.1 cells was showed by overlaying enhanced darkfield microscopy images (AuNPs pseudo-colored yellow) onto fluorescent microscopy images of cells stained for actin (cyan) and cell nuclei (magenta). Scale bars represent 10 μm.

**Figure 3:**
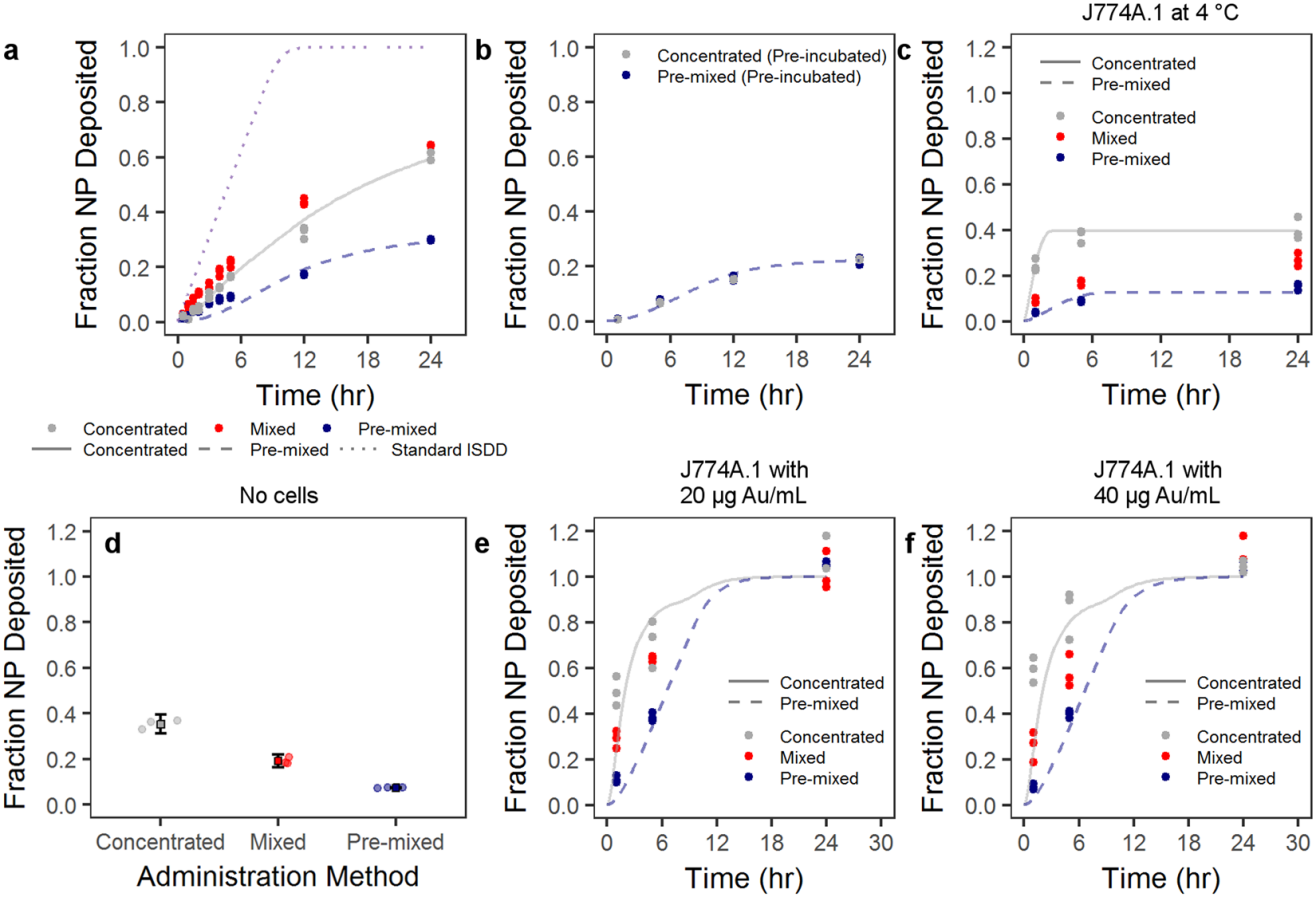
Particle cell interaction is dependent on administration method. (**a**) Particles administered as a concentrated, mixed, or pre-mixed dose showed variable uptake by J774A.1 mouse monocyte/macrophages as determined by ICP-OES. (**b**) This phenomenon was mitigated by first incubating the particle at a higher concentration in complete cell culture media, and then either adding as a concentrated dose or pre-mixing (pre-incubation). The trend in administration-dependent cellular interaction was conserved over various scenarios. (**c)** Administration at 4 °C was conducted to reduce active cellular uptake. (**d**) Non-specific NP adsorption to the culture plate was tested by incubating particles in complete cell culture medium overnight. Circles represent single data points and boxes represent mean ± one standard deviation. (**e**,**f**) AuNP-PVP deposition at 20 and 40 μg Au/mL, respectively, was tested over 24 hr. Lines indicate modified ISDD fit of data with concentrated or pre-mixed assumptions, as well as the standard ISDD conditions.

We studied the effect of the NP administration methodology on cellular uptake by J774A.1 mouse monocyte/macrophages. Cells were exposed to AuNP-PVP via the concentrated, mixed, or pre-mixed methods for varying time points. J774A.1 cells were chosen as a model cell line because macrophages are phagocytic cells which readily uptake NPs^39–41^. These cells were therefore believed to give the most consistent conditions that would fulfill the ISDD model boundary conditions (i.e. the bottom of the cell culture dish is treated as a NP sink due to rapid particle uptake). At varying time points over 24 hr of exposure, AuNP-PVP uptake or deposition by J774A.1 cells was visualized by fluorescent and dark field hyperspectral microscopy (Fig. 2), and quantified via inductively coupled plasma-optical emission spectroscopy (ICP-OES). The deposition of AuNP-PVP following administration via the three methods showed that the fraction of the initial dose deposited for concentrated, mixed, and pre-mixed after 24 hr was 0.61, 0.63, and 0.30, respectively (Fig. 3a). It is clear that the administration method influences cellular association of NPs even from the early time points of the experiment. However, it was unclear what drives this effect.

To further investigate the potential effect of administration on the surface of the NP, we pre-incubated the NPs at an intermediate concentration (e.g. 700 μg Au/mL) in cDMEM prior to administration via the concentrated or pre-mixed methods (Fig. 3b). By doing this pre-incubation any differences due to, for example protein adsorption, might be mitigate and the only difference between the two methods is thus physical. Indeed, it is apparent that the influence of administration was completely mitigated after the pre-incubation, thus indicating an effect of administration on the “biological identity” of the NPs.

It was interesting to see that the pre-mixed administration resulted in less particle deposition compared to the concentrated or mixed methods. In order to understand the results of these experiments better, we performed calculations based on the ISDD model (a full description of the calculations can be found in the Supporting Information).

The ISDD model makes several assumptions and sets boundary conditions regarding particle deposition and uptake: cells in the dish form a confluent layer, particles are stable or form “stable aggregates”, the bottom of the well-plate is treated as a particle sink (i.e. particles reaching this space are adsorbed or taken up by cells), and, importantly, that NPs are uniformly distributed in the cell culture media. Generally these are fair terms for an *in vitro* experiment, however as we show, these conditions are not always possible depending on the experiment. Thus, we altered the model to reflect some experimental differences. For one, we altered the boundary conditions so that the rate at which particles are uptaken by the cells is proportional to the concentration right outside the cells, and to the difference between the concentration of particles in the cells and a maximum concentration. This means that the rate at which particles can enter the cells depends on the particles surface and that cells can be saturated with cells. Moreover, the exists a stratified layer within the particle solution when adding via the concentrated method. Indeed, we observed that the particles formed a stratified layer when administered in the concentrated method (Fig. S2).

However, it is apparent that NP-cell interactions are affected by the way NPs are administered to cells. The environment surrounding NPs when introduced into complex biological media mediates this phenomenon, and concentration gradients in solution drive these protein-particle and inter-particle behaviors. This behavior may also be driven by the physico-chemical properties of NPs, which affect protein-protein, protein-NP, and NP-NP interactions. It was unclear whether cellular phenomena (e.g. active cellular uptake), NP concentration effects, or non-specific adsorption of AuNPs to the cell surface and well-plate bottom drove the differences in deposition and uptake for the AuNP-PVP. Therefore, we performed a number of experiments to observe whether active uptake, NP adsorption to the well plate, or NP concentration influenced our measurements. We investigated the role of active cellular uptake by observing AuNP-PVP uptake at a concentration of 70 μg Au/mL and at 4 °C (Fig. 3c). By conducting the experiments at lower temperature, receptor-mediated (active) cellular uptake would be mitigated and any deposition should arise from non-specific NP adsorption to cells, well-plate sticking, or diffusion across the cellular membrane^42,43^. The trend remained that concentrated and mixed methods deposited more compared to pre-mixed. This trend appears to be a non-specific phenomenon, whereby administration method dictates the (non-specific) adsorption of NPs to cells or the well-plate surface. Figure 3d shows the fraction of NPs adsorbed onto a well-plate that did not contain any cells following incubation of AuNP-PVP overnight at 37 °C in cDMEM. A similar deposition trend (concentrated > mixed > pre-mixed) was observed, at values similar to the maximum deposition at 4 °C and 24 hr in the presence of cells.

Further experiments conducted at lower concentrations (20 and 40 μg Au/mL, 37 °C) showed the same trend where concentrated and mixed administration deposited more than the pre-mixed (Fig. 3e,f). For both of these lower concentrations, the previous trend observed at earlier time points remained: Concentrated and mixed administration resulted in more AuNP-PVP deposition compared to the pre-mixed condition. However, at 24 hr it was shown that there were no differences between any of the three administration methods. These data show that regardless of NP concentration the deposition behavior is constant based on administration method. This indicates that the observed phenomenon is not linked to cellular behavior (e.g. active uptake), but is a product of the NP properties in solution.

By running the model for all the particles concentration (70, 40 and 20 μg Au/mL, 37 °C), it clearly appears that one at the highest concentration (70 μg Au/mL) there is a need to impose a maximum concentration of particles that the cells can uptake. In fact, these experiments are the only ones where after 24 hours only 30 to 60% of the particles have been taken up by the cells, compared to 100% for the lower concentrations. The conventional ISDD model, which imposes that all particles reaching the cells surface are taken up by them, substantially overestimates the uptake of particles in the case of 70 μg Au/mL. One can also observe, in the 20 and 40 μg Au/mL, that the standard ISDD model cannot account for the higher uptake of particles in concentrated case than in the premixed case. Since aggregation of particles has been ruled out, the only way to explain such a higher uptake is to consider a layering effect, where upon administration of a concentrated bolus of particles a thin layer with a concentration of particles about 10 times larger than that in the rest of the solution accumulates close to the cell surfaces and explains the faster increase in particles uptake compared to the ISDD model.

### Initial protein adsorption drives administration-dependent particle uptake

**Figure 4:**
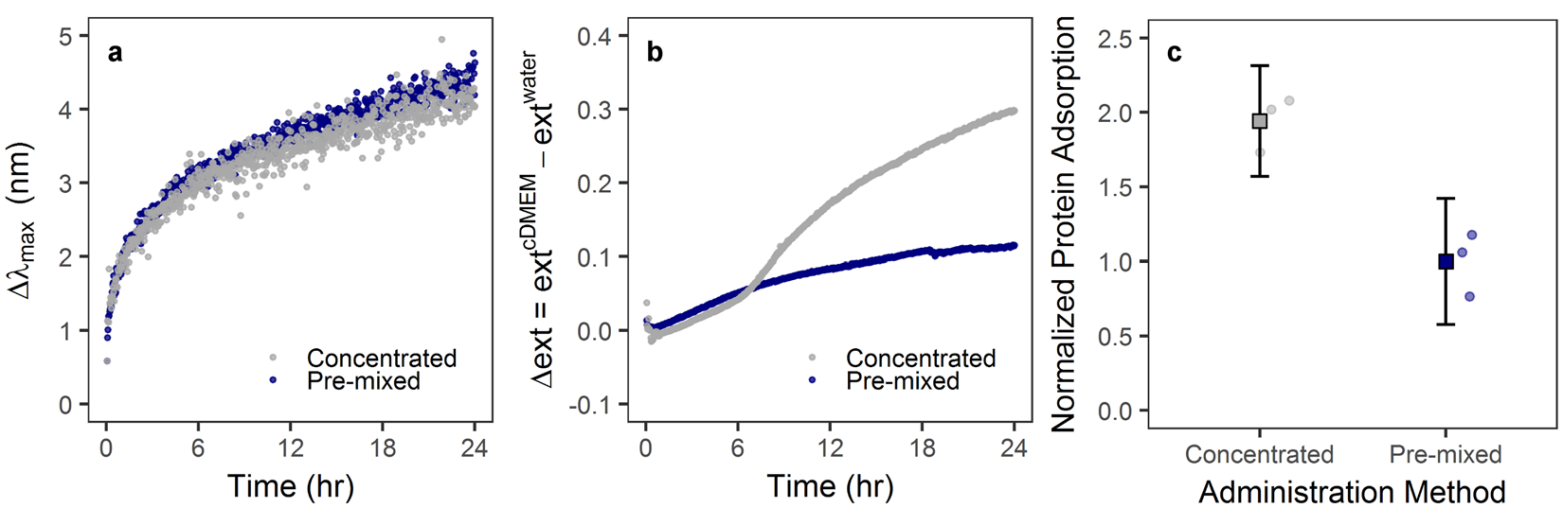
Particle introduction into complete cell culture media mediates protein adsorption to the particle surface. (**a**) UV-Vis analysis of AuNP-PVP over 24 hr showed a change in peak intensity wavelength. (**b**) UV-Vis analysis further showed a change in extinction intensity (Δext) dependent on administration method. These effects are indicative of protein adsorption to the nanoparticle surface. (**c**) Analysis of protein adsorption showed that, after 24 hr incubation in complete cell culture medium, AuNP-PVP administered via the concentrated route had an approximately 2-fold increase in protein adsorption compared to nanoparticles administered via the pre-mixed route.

In a previous work by Lesniak *et al*.^44^, it was shown that the presence of a protein corona can limit or slow particle adsorption to cells/surfaces in serum-free media. Thus, the protein corona can mediate the “adhesion” of particles on the cellular surface, and our observed difference in cellular deposition can therefore be supported by the variation of protein adsorption due to administration method. This hypothesis was investigated by following the change of extinction intensity in UV-Vis following different administration methods–higher protein adsorption was observed for concentrated compared to pre-mixed administration (Fig. 4a,b).

Some studies have revealed that particle uptake was enhanced in the presence of serum proteins on polystyrene NPs^45,46^, and Prapainop *et al*.^47^ also demonstrated that the coating layer on the NP surface can lead to misfolding of corona proteins, which then triggered NP uptake by specific cells that otherwise would not have done so. This protein structure modification may occur partially here because it has been demonstrated that some proteins in the presence of PVP showed higher refolding rate and aggregation-prone intermediates^48,49^. However, a slight red-shift of the LSPR band (max = 4–5 nm) was observed in AuNPs due to the formation of protein corona on particle surface (Fig. 4a). A similar red-shift for BSA adsorption on polymer-coated gold nanorods was reported previously by Boulos *et al*.^50^. These spectroscopic observations were in agreement with the change of hydrodynamic size measured under the same conditions by DDLS (Fig. 1d). The hydrodynamic size showed an increase of approximately 30 nm for AuNP-PVP, confirming the formation of protein corona, which might be composed of multiple protein layers. Most plasma proteins present a hydrodynamic diameter around 3–15 nm. Therefore, the protein corona in our case follows the model proposed by Simberg *et al*.^51^–a primary protein layer that adsorbs to the NP surface directly and a secondary layer that associates with the primary layer via protein-protein interactions. This multiple layers configuration may alter the activity of the proteins in the primary layer or modify the physiological response of the NPs, which may have an impact in the cellular uptake studies.

Interestingly, an increase in the extinction intensity over time was also observed (Fig. 4b). This change of the extinction can also be attributed to protein adsorption on the NP surface, since the intensity also depends on changes in the complex dielectric function near the interface between the metal and the media. Nath and Chilkoti^52^ and Shin *et al*.^53^ demonstrated that the quantification of the protein adsorption/binding to AuNPs is comparatively facile using the measurement of extinction intensity. In these reports the intensity of maximum extinction increased as the increase in the concentration of protein bonded on gold surface. In addition, Nath and Chilkoti^52^ reported that 12 nm diameter AuNPs can detect a refractive index change through the extinction intensity up to 24 nm away from the NP surface, whereas larger AuNPs can detect refractive index changes at a distance greater than 40 nm. Therefore, we can extract qualitative information about the amount of proteins adsorbed on NPs surface through the change of the extinction intensity upon incubating them in cDMEM.

The administration method-dependent response of AuNP-PVP, monitored in real time, is shown in Fig. 4b. The change of the extinction intensity was obtained with respect to the extinction in water (no presence of proteins). The change of the extinction profiles show a pronounced rise in the extinction intensity upon concentrated administration, while only a slight increase of the intensity for AuNP-PVP was detected upon pre-mixed administration. This finding demonstrates that the *in vitro* methodology can affect how many proteins are adsorbed on the NP surface, varying the physico-chemical properties of the NPs. In turn, this can have consequences on the NP-cell interaction. In order to support the extinction measurements, we added AuNP-PVP to cDMEM via the pre-mixed or concentrated method. After 24 hr, we centrifuged the AuNP-PVP and washed them in PBS. We estimated the concentration of protein adsorbed to the NP surfaces with SDS-PAGE and AgNO_3_ staining using FIJI image analysis software to measure band density for every molecular weight at a fixed gold concentration (as determined by ICP-OES). Protein adsorption was compared by normalizing by the pre-mixed administration method (Fig. 4c). Strikingly, protein adsorption with concentrated administration was 2-fold higher than with the pre-mixed method, even 24 hr after initial incubation. This indicates that initial administration has a lasting effect on particle biomolecular corona and subsequent cellular interaction.

### Surface coating and size are critical factors mediating nanoparticle uptake

**Figure 5:**
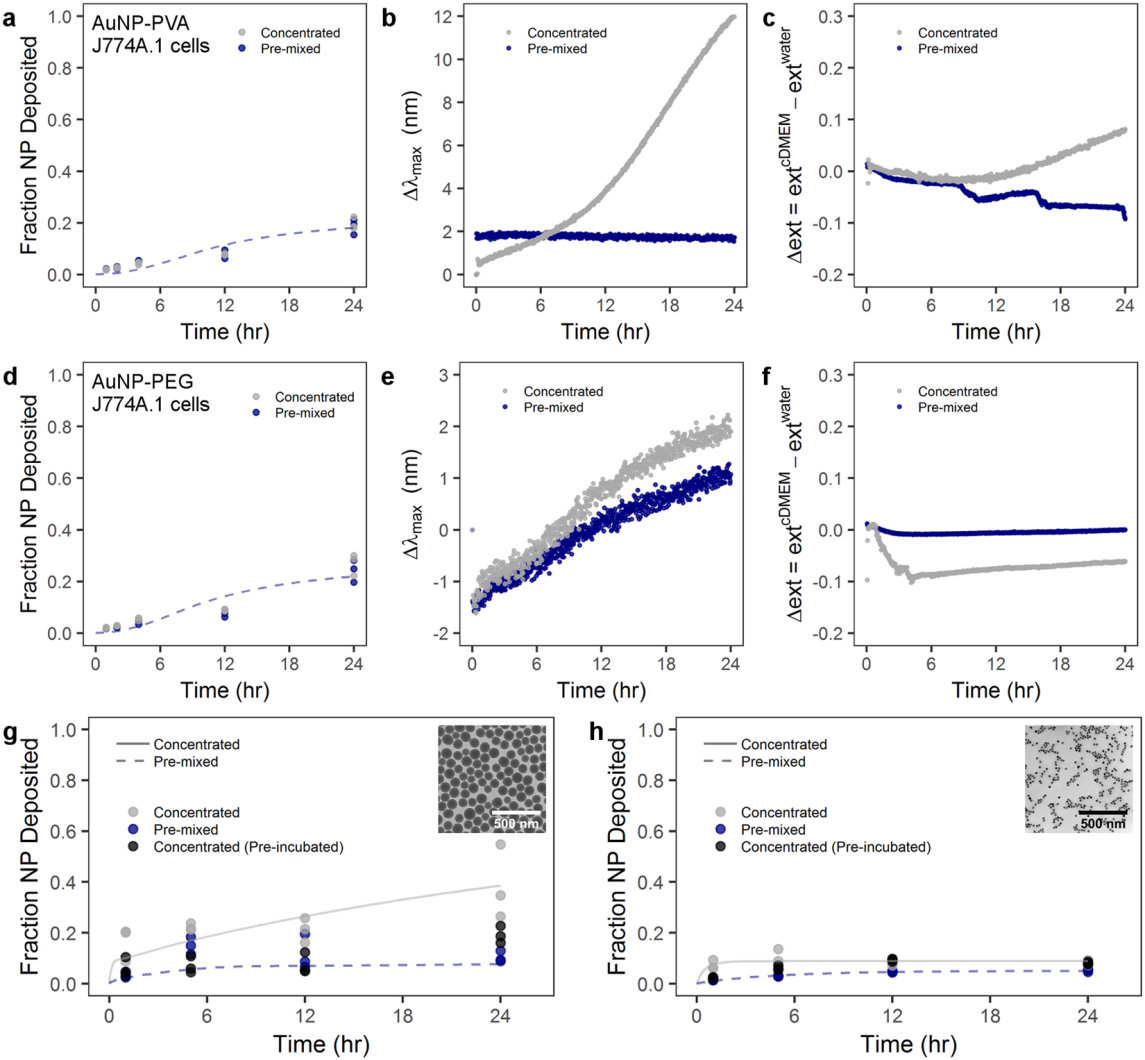
Administration method effect on particle-cell interaction is heavily dependent on particle material, size, and surface coating. Cellular uptake by J774A.1 murine cells and UV-Vis analysis of (**a**–**c**) PVA-coated AuNPs and (**d**–**f**) PEG-coated AuNPs. J774A.1 uptake of (**g**) PVP-coated SiO_2_ NPs and (**h**) PVP-coated superparamagnetic iron oxide NPs. (**b**,**e**) LSPR band shift for PVA-coated and PEG-coated AuNPs, respectively, following the concentrated or pre-mixed administration. (**c**,**f**) Time-dependent change of the extinction intensity (Δext) with respect to the extinction in water at maximum for PVA-coated and PEG-coated AuNP, respectively. Lines indicate modified ISDD fit of data with concentrated or pre-mixed assumptions. Inset TEM micrographs show (**g**) PVP-coated SiO_2_ and (**h**) PVP-coated superparamagnetic iron oxide NPs. Scale bars represent 500 nm.

NP behavior in solution and with cells will be a factor of, among other things, the particle size, surface properties, and surface functionality. For example, PVP-coated AuNP will behave differently in solution compared to, for example, PEG-coated AuNP. Therefore, two AuNP formulations were synthesized as a control with amine-terminated PEG and amine-terminated poly(vinyl alcohol) (PVA) coatings. Both formulations showed colloidal stability over 24 hr in cDMEM independent of administration method (Fig. S3). In a control experiment, PVA-coated or PEG-coated AuNP with a core diameter of 116 nm were administered to J774A.1 mouse monocyte/macrophages via the pre-mixed and concentrated methods (Fig. 5a,d, respectively). We showed that there was no difference between the pre-mixed and mixed administration methods with PVA-coated or PEGylated AuNP. Moreover, there was significantly less NP uptake with PVA or PEG-coated AuNP compared to PVP-coated AuNP. The simulation of these data indicate that also in this case the standard ISDD boundary condition is not be able to account for the observed behavior. Only by imposing a substantial reduction in the rate of uptake of the particles the data can be properly simulated.

This significant difference in uptake between PVP-coated AuNP and PEGylated or PVA-coated AuNP is explained by the “stealth” effect^22,54–56^. In essence, PEGylation reduces protein adsorption to NP and consequently NP are less prone to be recognized and phagocytosed by cells. Rather, the cells “see” water due to the mitigation of protein adsorption and the flexible nature of these hydrophilic polymer chains. This hypothesis was supported for the spectral evolution acquired by UV-Vis spectroscopy for PVA-coated and PEGylated AuNPs. Figure 5b,e shows no observable red-shifted of the LSPR band or increase of extinction intensity except in the case of AuNP-PVA(NH_2_) with concentrated administration, where there is a noticeable variation in the maximum wavelength after of 8 hr. However, the hydrodynamic sizes of all NPs did not show any appreciable increase (Fig. S3), demonstrating that both polymer coatings prevent the protein adsorption within 24 hr. The variation in the spectral properties of AuNP-PVA(NH_2_) can be likely attributed to hydrogen bonding and hydrophobic processes to enhance the colloidal stability in cDMEM, since the hydrodynamic size in water (232 nm) is 2-fold bigger than in cDMEM (136 nm).

In order to probe the effects of particle size and density, we studied PVP-coated SiO_2_ and PVP-coated superparamagnetic iron oxide NPs (PVP-SPIONs) with core diameters of 99 ± 9 and 22 ± 1 nm, respectively (Fig. 5g,h inset TEM images and Fig. S4). The fraction of deposited SiO_2_ NPs was determined by ICP-OES measurements, was similar to the AuNP-PVP case–concentrated administration resulted in greater NP deposition at 24 hr compared to pre-mixed administration (Fig. 5g). Furthermore, by pre-incubating 100 nm PVP-coated SiO_2_ at a higher concentration in cDMEM and then administering via the concentrated method (i.e. pre-incubation) there was no difference compared to the conventional pre-mixed case. However, size seems to play an important role, because size will affect the sedimentation behavior of particles^24^. The small, 22 nm PVP-SPIONs showed no difference between the concentrated, pre-mixed or concentrated with pre-incubation scenarios (Fig. 5h). A similar protein study showed that for both PVP-coated SiO_2_ NPs and PVP-SPIONs, the concentrated administration resulted in more protein adsorption (approximately 20 and 50% more, respectively) compared to pre-mixed administration at 24 hr after introduction to media. This presumably points to an interplay between protein adsorption onto particles, due to administration methods, and the particle size/density dictating sedimentation velocity (Fig. S5).

## Conclusion

In order to advance our understanding of nanoparticle interactions with biological systems, it is pertinent that we fully understand how NPs behave in an *in vitro* environment including the application method and behavior in cDMEM over time. When trying to compare *in vitro* results between laboratories, there must be a comprehensive understanding of several key parameters: NP physico-chemical properties, colloidal stability in biological media, experimental design and implementation, and analytical methods. These factors are critical if we are to move forward in our understanding of nanotoxicology, particle-cell behavior, and use of particles for biomedical applications.

Here we have shown that a minor detail, the way in which NP are added to the cell culture, can have significant downstream effects in what we observe for the cellular interactions. Particles added as a concentrated “bolus” or as a bolus that is then mixed via pipetting, are more interacting with cells when compared to when NPs are pre-mixed to the desired concentration and then added to cells. The differences observed were also not attributable to active cellular mechanisms, but are more likely due to the initial introduction of NP to complex biological media. As a community, we should therefore make robust analytical efforts when interpreting *in vitro* data. This is of course non-trivial and requires a multidisciplinary approach. However, only with such precautions can we hope to gain a complete picture of NP-cell interactions.

## Supplementary information

Supplementary Information

## Data Availability

The data that support the findings of this study are available from the corresponding author upon reasonable request.
