# Supplementary material for: Nanoparticle administration method in cell culture alters particle-cell interaction
Source: Sci Rep. 2019 Jan 29;9:900. doi: 10.1038/s41598-018-36954-4 (PMC6351679; doi:10.1038/s41598-018-36954-4)
Supplement: Supplementary file 1 — Supplementary Information [file 41598_2018_36954_MOESM1_ESM.pdf]

Supporting Information

Nanoparticle administration method in cell culture alters particle-cell interaction

Thomas L. Moore[1], Dominic A. Urban[1], Laura Rodriguez-Lorenzo[1], Ana Milosevic[1], Federica Crippa[1], Miguel Spuch-Calvar[1], Sandor Balog[1], Barbara Rothen-Rutishauser[1], Marco Lattuada[2], and Alke Petri-Fink[1,2,*]

[1]Adolphe Merkle Institute, Université de Fribourg, 1700 Fribourg, Switzerland.
E-mail: alke.fink@unifr.ch

[2]Department of Chemistry, Université de Fribourg, 1700 Fribourg, Switzerland.

Contents

Experimental Methods **1**
Materials . . . 1
Nanoparticle synthesis and coating . . . 1
Nanoparticle characterization . . . 2
Cell culture . . . 3
Nanoparticle uptake and deposition . . . 3
Dual enhanced darkfield and fluorescent microscopy . . . 4
Protein adsorption on particles . . . 4
Mathematical model to describe nanoparticle deposition . . . 5

Supplementary Results **7**
Nanoparticle characterization . . . 7
Particle sedimentation . . . 7
Stability of PVA- and PEG-coated 116 nm AuNPs . . . 8
SiO_2 and SPION characterization . . . 8
SDS-PAGE and Protein Adsorption on Particles . . . 9

Bibliography **10**

Supplementary Files and Descriptions

Experimental Methods

Materials

Acetone, (3-aminopropyl) triethoxysilane (APTES), dichlorobenzene, diethyl ether, dimethyformamide, dimethylsulfoxide, gold(III) chloride trihydrate ($HAuCl_4$), hydroxylamine hydrochloride (NH_2OH), iron (III) chloride hexahydrate ($FeCl_3 \cdot 6\ H_2O$), 65% nitric acid (HNO_3), oleic acid, potassium hydroxide (KOH), silver nitrate ($AgNO_3$), sodium citrate, and tetraethyl orthosilicate (TEOS) were supplied by Sigma-Aldrich. Aqueous gold standard was supplied by Fluka. Polyvinylpyrrolidone (PVP) was supplied by Alfa Aesar GmbH. Hydrogen peroxide (H_2O_2) and sulfuric acid were supplied by Reacto Lab SA. Sodium oleate and trioctylamine were supplied by TCI. Cyanine5-NHS was supplied by Lumiprobe. All reagents were used as received without further purification. Sterile phosphate buffered saline (PBS) was supplied by Life Technologies. 37% hydrochloric acid (HCl) was supplied by Honeywell AG.

Nanoparticle synthesis and coating

Gold nanoparticle synthesis. Gold nanoparticles (AuNP) of were synthesized following previously described methods.[1] An aqueous solution of $HAuCl_4$ (500 mL, 0.5 mM) was brought to boil for 10 min in a 1 L Erlenmeyer flask. A sodium citrate solution (25 mL, 34 mM) was heated to 60 °C and quickly added to the gold salt solution. The solution was kept at a temperature of 100 °C with stirring for 20 min before the flask was removed from heat and left to cool to room temperature for 3 hours. To make the larger particles, an aqueous solution (100 mL) of $HAuCl_4$ (0.25 mM) and NH_2OH (1.96 mM) was prepared in a glass bottle, and homogenized with a magnetic stirrer. The previously prepared gold seed suspension was added (2.5 mL, 11.3 μM) and the mixture was stirred constantly for approximately 20 min. This resulted in nanoparticles with a diameter of approximately 45 nm. A second growth step followed, however the freshly prepared 45 nm particles were used instead of the seed solution (35 mL), and the reaction volume was increased to 400 mL. This resulted in approximately 100 nm particles.

PVP, MW = 8 kDa, was coated electrostatically to the AuNP surface. AuNP (200 mL, 273 μM Au) were added dropwise to a solution of PVP in Milli-Q water (5 mL, 1.4 mM) under constant stirring. The solution was stirred for 24 hr, and free polymer was washed via centrifugation in Milli-Q water three times at 4,000 g for 20 hr. All samples were stored at 4 °C protected from light.

NH_2-PVA-SH coating of AuNPs. NH_2-PVA-SH was synthesized following a method previously described by Kinnear, et al.[2] A total of 150 mL of 116 nm AuNPs ([Au] = 0.25 mM) was added dropwise to a solution of NH_2-PVA-SH solution (4.1 mg) at pH 6 under shaking. The mixture was then left to react overnight. The particles were centrifuged at 1,000 g for 15 min and re-dispersed in water.

Superparamagnetic iron oxide nanoparticle synthesis. Superparamagnetic iron oxide nanoparticles (SPIONs) were synthesized following a thermal decomposition reaction.[3,4] An iron-oleate complex was formed by dissolving 8.66 g (32 mmol) $FeCl_3$ and 29.792 g (97.8 mmol) sodium oleate in a solution of 64 mL ethanol, 48 mL distilled water, and 112 mL hexane. The reaction was stirred at 70 °C for 4 hr under reflux. The organic phase was then washed three times with 50 mL distilled water in a separatory funnel, and the hexane was evaporated. The iron oleate complex was stored in at 4 °C until further use.

Iron oxide nanoparticles were synthesized by dissolving 14.84 g of the iron-oleate complex with 2.37 mL of oleic acid in 107.7 mL of trioctylamine, and degassing at 50 °C for 2 hr. This solution was heated to 320 °C under argon with a 2-step heating process: First increasing to 120 °C at 10 °C/min, and then heating to 320 °C at 3 °C/min. The reaction continued for 1 hr. The reaction was cooled to room temperature (or 25 °C) and an excess of hexane and ethanol was added to precipitate the nanoparticles. Centrifugation at 30,000 g was used to separate the iron oxide nanoparticles. NP were suspended in hexane.

SPIONs dispersed in hexane were transferred to an aqueous phase via a ligand-exchange process.[5] 74 mg of SPIONs in hexane were precipitated in 50 mL of ethanol and separated magnetically for 2 days. Then SPIONs were re-dispersed in 20 mL of dichlorobenzene and dimethyformamide (ratio 1:1). 61.6 mg of citric acid were added and the solution was kept at 100 °C for 24 hr under magnetic stirring. The day after the SPIONs were washed by first precipitating in 40 mL of diethyl ether and recovering with a magnet. Next particles were washed three times in acetone (50 mL) and recovered with magnet. Finally SPIONs were re-dispersed in 5 mL of water and 3 drops of NH_3 are added to stabilize the suspension.

Silica nanoparticle synthesis. 100 nm silica NPs (SiO_2 NPs) were synthesized following a modified Stöber approach.[6,7] First, an NHS-functionalized Cyanine5 dye (Cy5, 2.3 mg, 3.7 µmol) was attached to (3-aminopropyl) triethoxysilane (APTES, 3 µL, 12.8 µmol) silica precursor. Cy5 was dissolved in 200 µL dimethylsulfoxide with stirring, and then APTES was added and allowed to stir overnight protected from light. Product was used the next day without any further purification.

To synthesize fluorescent SiO_2 NPs, 58 mL diH_2O, 162 mL ethanol, and 7.8 mL of 28% NH_4OH were mixed together in a large, round-bottom flask. The solution was heated to 70 °C with stirring. Into this solution, 22 mL (98.5 mmol) of tetraethyl orthosilicate were added and allowed to stir for 2 minutes. Afterwards, 200 µL of APTES-Cy5 solution were added, and the solution was stirred for 4 hr. The reaction was then removed from heat and allowed to cool to room temperature. The SiO_2 NPs were dialyzed for 1 week in diH_2O with gentle stirring.

Nanoparticle characterization

Transmission electron microscope (TEM) images were acquired using an FEI Tecnai Spirit TEM with a Veleta 2048 $\times$ 2048 wide angle CCD camera at 120 kV. Size distribution was measured using FIJI particle analysis software (National Institutes of Health, USA, ImageJ 1.51h).[8]

UV-Vis spectroscopy was recorded on a Jasco V-670 spectrophotometer at 25 °C, using 10 mm path length quartz cuvettes. As a first approximation, the particle size was determined directly from UV-Vis spectra using the tabulated theoretical data of uncoated spherical AuNPs in water described in Haiss et al.[9] For kinetic measurements, NP were added to complete, phenol red-free Dulbecco's Modified Eagle's Medium (DMEM; supplemented with 10% fetal bovine serum and 1% penicillin-streptomycin) via the pre-mixed or concentrated methods, and measurements were conducted at 37 °C for 24 hr with a measurement made every 2 minutes.

Hydrodynamic size of NPs was measured via depolarized dynamic light scattering (DDLS). Samples were diluted into complete, phenol red-free DMEM and kinetic measurements were made in media

every three minutes over 24 hr at 37 °C and a scattering angle of 30°. DDLS spectra were collected using a commercial goniometer instrument (3D LS Spectrometer, LS Instruments AG, Switzerland). The primary beam was formed by a linearly polarized and collimated laser beam (Cobolt 05-01 diode pumped solid state laser, λ= 660 nm, P max. = 500 mW), and the scattered light was collected by single-mode optical fibers equipped with integrated collimation optics. The collected light was coupled into two high-sensitivity APD detectors via laser-line filters (Perkin Elmer, Single Photon Counting Module), and their outputs were fed into a two-channel multiple-tau correlator. The signal-to-noise ratio was improved by cross-correlating these two channels. With respect to the primary beam, depolarized scattering was observed via cross-polarizers. The incoming laser beam passed through a Glan-Thompson polarizer with an extinction ratio of 10^{-6}, and another Glan-Thompson polarizer, with an extinction ratio of 10^{-8}, was mounted in front of the collection optics.

Cell culture

J774A.1 mouse monocyte/macrophages were supplied by ATCC. Dulbecco's Modified Eagle's Medium (DMEM), fetal bovine serum (FBS) and penicillin-streptomycin were supplied by Gibco. Cells were grown in DMEM supplemented with 10% FBS and 1% pen-strep at 37 °C and 5% CO_2.

Nanoparticle uptake and deposition

Cells were seeded at 62,500 cells per well (65,790 cells/cm^2) in 48-well plates. Cells were allowed to attach overnight, and the next day media was removed and cells exposed to AuNP-PVP with three administration methods: (1) Pre-mixed. NPs were diluted to 70 µg/mL in complete DMEM, all media was removed from wells and then replaced with the pre-mixed NP solution. (2) Mixed. All media was removed from wells and replaced with fresh complete DMEM. A concentrated volume of NPs was added to the wells, bringing the final volume to a concentration of 70 µg/mL. This solution was mixed by gently pipetting three times. (3) Concentrated. All media was removed from wells and replaced with fresh complete DMEM. A concentrated volume of NPs was added to the wells, bringing the final volume to a concentration of 70 µg/mL. This solution was left undisturbed. All final volumes in the wells were 500 µL. Cells were incubated with NPs for various time points and under the various conditions, and at the end of each time point wells were washed twice with phosphate buffered saline (PBS).

ICP-OES quantification AuNP association/uptake by cells. AuNP deposition/uptake by cells was measured by inductively coupled plasma-optical emission spectroscopy (ICP-OES) on an Optima 70000 DV (Perkin Elmer). Cells were treated with a 2:1 mixture of 65% HNO_3 and 30% H_2O_2 at 300 µL per sample, and digested for several hours. The samples were briefly sonicated prior to the addition of 400 µL of 37% HCl and digested overnight. Finally, the samples were diluted ten-fold with Milli-Q H_2O. The treated samples were then measured by ICP-OES at a wavelength of 242.795 nm for gold. A standard curve of aqueous gold solutions, in an equivalent sample matrix, was recorded to quantify the amount of cell-associate gold.

ICP-OES quantification SPIONs association/uptake by cells. Upon SPIONs incubation, cells were treated with a 2:1 mixture of 65 % HNO_3 and 30 % H_2O_2 at 300 µL per sample and digested for 4 hr at 40 °C. The samples were sonicated at 50 °C for 15 min prior to the addition of 400 µL of 37 % HCl and digested overnight at 37 °C. Finally, samples were collected in 15 mL Falcon tubes and diluted ten-fold with Milli-Q water. NP association/uptake by cells was measured by ICP-OES on a Perkin Elmer Avio 200. ICP-OES measurements were performed at a wavelength of 238.204 nm (limit

of detection (LOD) = 0.13 µg/L and limit of quantification (LOQ) = 14 µg/L), and at an axial plasma view. The plasma flow was 8 mL/min and the sample flow rate 1.5 mL/min. A washing-step of 10 % HNO_3 was performed between each measurement and each sample was measured three times.

ICP-OES quantification of SiO_2 association/uptake by cells. Upon SiO_2 NPs incubation, cells were kept at -80 °C until further processing. NP association/uptake by cells was measured by ICP-OES on a Perkin Elmer Avio 200. The thawed cells were treated with 1 mL of 0.5 M KOH, and pre-digested overnight at 37 °C. The pre-digested samples were collected by scrapping the cells and transferred into a PTFE-TFM vessels (HVT50, Anton Paar) and the volume filled to 3 mL. Digestion of the samples was conducted in a microwave Multiwave Pro (Anton Paar, Germany) equipped with a rotor 24HVT50. The maximum power and pressure were 1200 W and 20 bars. The microwave heating program was 200 °C for 7 min (7 min ramp) and 70 °C for 20 min for the cooling step. After cooling, the samples were acidified with 1 mL of 2.25 M sulfuric acid and transferred then to 15 mL Falcon tubes. Finally, the samples were diluted 5-fold with 1% HCl and added 100 µL of 25 µg/mL of Y in 2% HCl as an internal standard. The treated samples were measured by ICP-OES at a wavelength of 251.611 nm (silicon; limit of detection (LOD) = 24 µg/L and limit of quantification (LOQ) = 79 µg/L) and 371.029 nm (yttrium), and at an axial plasma view. The plasma flow was 8 mL/mL and the sample flow rate 1.5 mL/min. A washing step with 2% sulfuric acid was performed between each measurement and each sample was measured three times.

Dual enhanced darkfield and fluorescent microscopy

Cells were seeded onto a chambered glass slide and exposed to AuNP-PVP at 70 µg/mL overnight at 37 °C and 5% CO_2. As a control, wells were examined containing media only (no cells), AuNP-PVP only (no cells), and cells only. After incubation, chambers containing cells were washed twice with PBS, and cells were fixed with 4% formaldehyde in PBS. Cells were stained with rhodamine-phalloidin (for F-actin) and DAPI (for cell nuclei). Cells were mounted in Glycergel mounting medium (Dako Schweiz GmbH, Baar, Switzerland) and cover-slipped. Dual enhanced darkfield and fluorescent microscopy images were acquired on a CytoViva enhanced darkfield microscope (CytoViva, Inc., Auburn, AL, USA) outfitted with a Dolan-Jenner DC-950 light source, UPL Fluorite 100× objective, SPECIM V10E imaging spectrograph with a PCO pixelfly detector, and Texas Red and DAPI emission filters. Images were acquired by taking a z-stack with 79 frames (with 500 nm between each z-position). Image post-processing was done using FIJI image analysis software.[8] A maximum intensity projection was taken for each channel, and the images were overlayed. Darkfield images were pseudo-colored yellow for the AuNPs, while the fluorescence channels were pseudo-colored cyan and magenta for F-actin and nuclei, respectively.

Protein adsorption on particles

Protein adsorption onto particles was evaluated using SDS-PAGE with $AgNO_3$ staining. Particles were added into 50 mL of DMEM supplemented with 10% fetal bovine serum via either the pre-mixed or concentrated methods. Particles were incubated overnight in complete media at 37 °C and the following morning they were collected via centrifugation (2,500 g for 20 minutes). Particles were then washed 2× in PBS to remove weakly bound proteins. Particle concentration was measured via both UV/Vis and ICP-OES and all samples were diluted to the same concentration. Next, samples were prepared for SDS-PAGE by diluting in a Laemmli reducing sample buffer and incubating for 48 hr at 75 °C. 40 µL of sample were loaded into a 4-15% gradient Mini-Protean® TGXTM precast gel (Bio-Rad, Hercules, CA, USA) and a Precision Plus Protein Dual Color™ (Bio-Rad) standard was used as a molecular weight marker. Gels were run with a Bio-Rad electrophoresis system: First, gels were run at 80 V to load the

proteins into the gel and then at 120 V to separate the proteins. Proteins were stained using an $AgNO_3$ protocol,[10] and imaged on a FluorChem E (ProteinSimple) system. Gel analysis was done using FIJI image analysis software. Briefly, each lane was analyzed and the area under the curve (AUC) for each protein band was measured for each formulation. The total intensity, represented by the sum of AUC for each formulation, was compared by normalizing by the pre-mixed condition (for each particle type).

Mathematical model to describe nanoparticle deposition

The modeling strategy followed in this work followed closely the ISDD model first proposed by Hinderliter et al.,[11] which is based on a sedimentation-diffusion equation describing the time and space evolution of the particles in a cell culture. In its original formulation, it is assumed that all particles arriving at the cell surface are taken up by the cells via endocytosis mechanisms. This physical effect is mathematically modelled by imposing as a boundary conditions that the concentration of particles at the bottom of a well-plate (i.e., in contact with the cells) is always zero. The second assumption typically made is that, initially, the concentration of particles is uniform in the entire well-plate. In order to quantitatively modeling the behavior of particles observed in this work, both aforementioned assumptions need to be relaxed. The relevant equations used are listed in the following:

$$\frac{\partial C}{\partial t} = D\frac{\partial^2 C}{\partial z^2} + V\frac{\partial C}{\partial z} \tag{1}$$

In Equation (1), $C(t,z)$ is the particle concentration, which is a function of time t and of the vertical spatial coordinate z in the well-plate, D is the particles diffusion coefficient and V is their sedimentation velocity. The latter quantities are respectively given by:

$$D = \frac{k_B T}{6\pi\eta R_H}, \qquad V = \frac{2g\Delta\rho R_P^3}{9\eta d_H} \tag{2}$$

Where k_B is Boltzmann constant, T the temperature, η the viscosity of the medium in which particles are dispersed, R_P is the particle core radius, R_H the particle hydrodynamic radius, g is gravity acceleration and finally $\Delta\rho$ is the difference between the particle and the medium densities. Depending upon the simulation performed, the initial concentration profile is given by:

$$C(t=0,z) = C_0$$

or

$$C(t=0,z) = \begin{cases} 10C_1 \text{ for } 0 \le z \le \frac{L}{10} \\ \\ C_1 \text{ for } \frac{L}{10} \le z \le L \end{cases} \tag{3}$$

The first case represents a uniform concentration profile, with concentration C_0, while the second case refers to a two layers model, with a bottom layer (with a thickness of about 1/10 of the total height

of liquid in the well-plate, equal to L), in close contact with the cells having a concentration 10 times higher than the one in the rest of the wall. The value of C_1 is chosen such that the total number of particles in the well is identical to the case of uniform concentration. The rationality behind the choice of a two layers model is the empirical observation that a particle administration in one concentrated bolus leads to the initial formation of two layers of particles, the bottom one having a much higher concentration of the rest. We chose the factor 10 because the particle concentration in the bolus is approximately 10 times higher than the final one in the well-plate.

We chose one of boundary conditions applied for the solution of the equation as follows:

$$D\frac{\partial C}{\partial z} + V \cdot C\Big|_{z=L} = 0 \tag{4}$$

These is the standard ISDD boundary conditions, expressing zero particle flux at the upper surface of the liquid in the well-plate, where the liquid is in contact with air. On the other hand, the second boundary condition has been modified compared to the standard ISDD model, in order to explain the variety of phenomena observed in this work.

Under some conditions we imposed a different boundary conditions at $z = 0$, i.e., that the total flux of particles taken up by the cells is fixed, and equal to k:

$$D\frac{\partial C}{\partial z} + V \cdot C\Big|_{z=0} = \frac{dn}{dt} = h \cdot C(z = 0, t)(n_0 - n) \tag{5}$$

This boundary condition assumes that the flux at the cell surface is equal to the rate of change of particles concentration in the cells, denoted by n. This rate of accumulation is assumed to be proportional to the concentration of particles in the solution at the boundary with the cells through a mass transfer coefficient h, but also to the difference between the concentration of particles in the cells and a maximum concentration n_0. In this manner the model can account for both the rate at which particle can be taken up by cells, and for the "saturation" of cells, i.e., the fact that particles in cells can only reach a maximum quantity.

The two parameters, i.e., the mass transfer coefficient h and the maximum concentration of particles in cells n_0 are treated as adjustable parameters. One should note that the boundary condition 5 reduces to the standard ISDD boundary condition when the value of the mass transfer coefficient h and the maximum concentration of particles in cells n_0 substantially exceeds the overall concentration of particles in administered to the cells.

Supplementary Results

Nanoparticle characterization

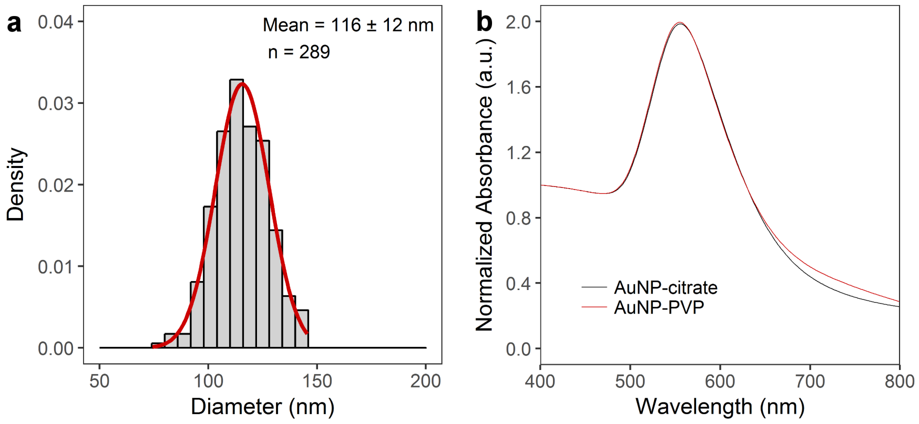

Figure S1. Characterization of 116 nm AuNP via transmission electron microscopy and UV-Vis spectroscopy. **a,** Histogram of AuNP core diameter as determined from image analysis shows a mean of 116 $\pm$ 12 nm. **b,** UV-Vis spectroscopy (showing NP before and after PVP-coating).

Particle sedimentation

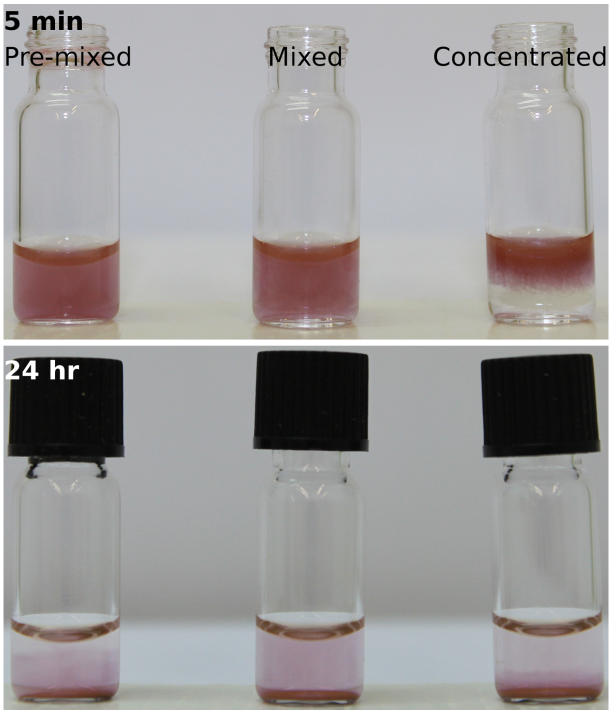

Figure S2. Photographs of 116 nm AuNP-PVP following the three different administration methods into phenol red-free cDMEM after 5 minutes and after 24 hr.

Stability of PVA- and PEG-coated 116 nm AuNPs

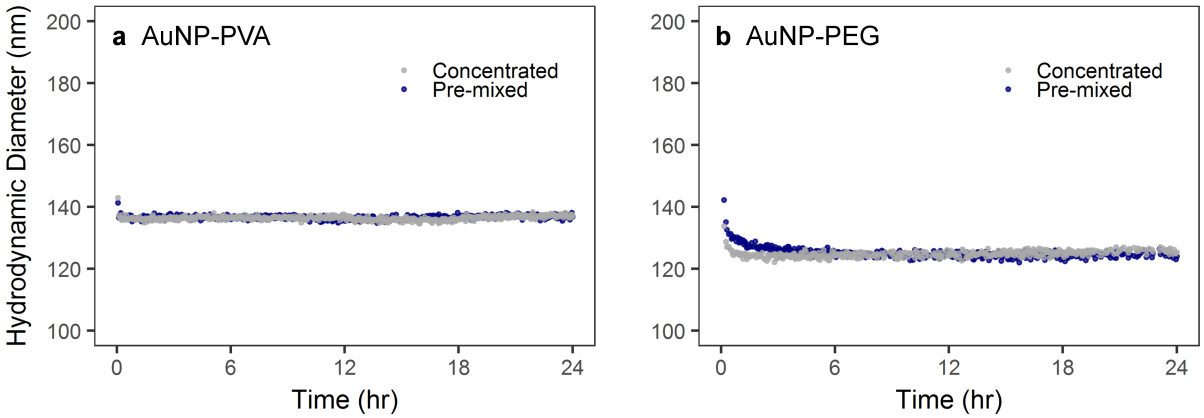

Figure S3. Depolarized dynamic light scattering (DDLS) measurements of (A) PVA-coated 116 nm AuNPs and (B) PEGylated 116 nm AuNPs over 24 hr in complete DMEM following pre-mixed or concentrated administration.

SiO_2 and SPION characterization

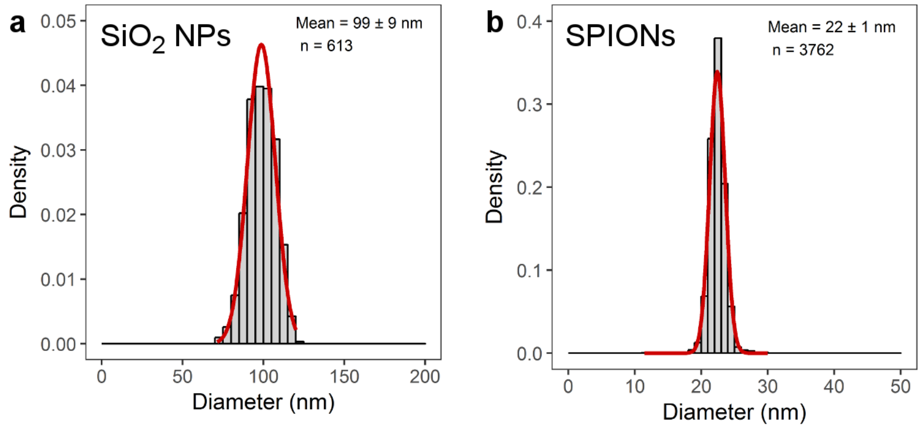

Figure S4. Size evaluation of SiO_2 NPs and SPIONs, as determined by transmission electron microscopy. a, Histogram of SiO_2 NPs showing a mean core diameter 99 ± 9 nm. **b,** Histogram of SPIONs showing a mean core diameter of 22 ± 1 nm.

SDS-PAGE and Protein Adsorption on Particles

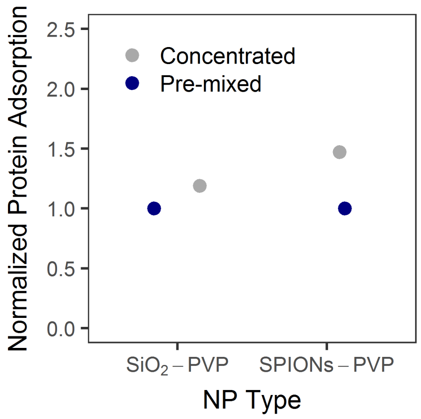

Figure S5. SDS-PAGE analysis of protein adsorption onto PVP-coated SiO_2 NPs and PVP-coated SPIONs after 24 hr incubation in cDMEM showed that in both cases, the concentrated administration resulted in higher protein adsorption.

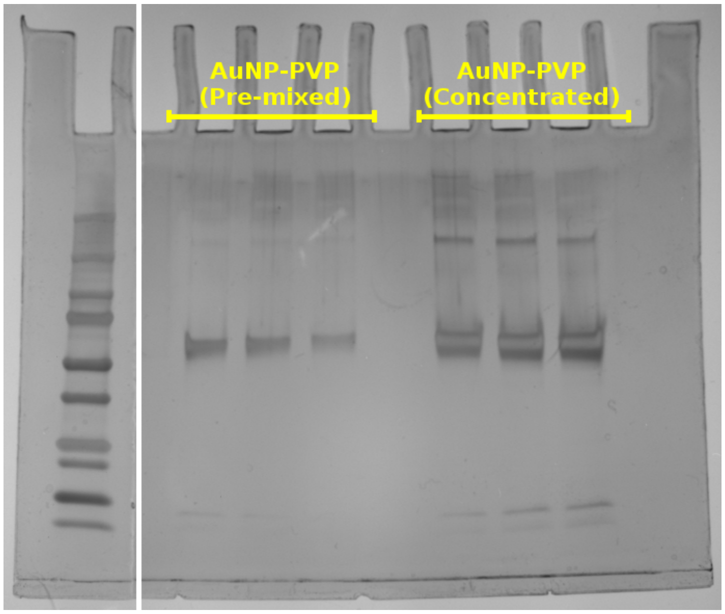

Figure S6. SDS-PAGE gel of AuNP-PVP incubated in complete media supplemented with 10% fetal bovine serum after administration via the pre-mixed or concentrated approach. Triplicates were done for each administration method. The first lane was cut from the gel prior to $AgNO_3$ staining to avoid over-development.

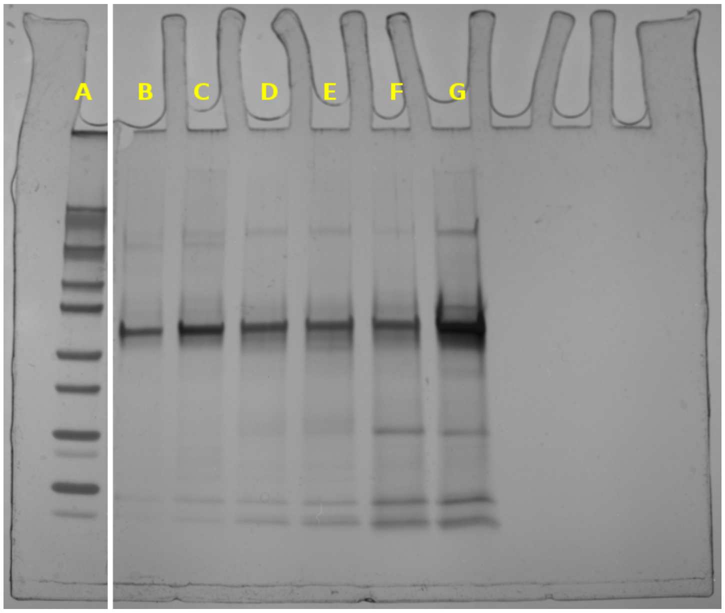

Figure S7. SDS-PAGE gel of different particles incubated in media supplemented with 10% fetal bovine serum. Particles were added into media via the pre-mixed or concentrated approach. (A) molecular weight ladder; (B) SPIONs-PVP, pre-mixed; (C) SPIONs-PVP, concentrated; (D) SiO_2 NP-PVP, pre-mixed; (E) SiO_2 NP-PVP, concentrated; (F) SiO_2 NP (uncoated), pre-mixed; (G) SiO_2 NP (uncoated), concentrated. The first lane, A, was cut from the gel prior to $AgNO_3$ staining to avoid over-development. The last two lanes, F and G, were not further investigated for this study because the particles were not stable in complete media.

Bibliography

1 Brown, K. R., Walter, D. G. & Natan, M. J. Seeding of colloidal Au nanoparticle solutions. 2. Improved control of particle size and shape. *Chem. Mater.* **12**, 306–313 (2000).

2 Kinnear, C. *et al.* Polyvinyl alcohol as a biocompatible alternative for the passivation of gold nanorods. *Angew. Chem. - Intl. Ed.* **53**, 12613–12617 (2014).

3 Park, J. *et al.* Ultra-large-scale syntheses of monodisperse nanocrystals. *Nat. Mater.* **3**, 891 (2004).

4 Sun, S. *et al.* Monodisperse MFe_2O_4 (M = Fe, Co, Mn) nanoparticles. *J. Am. Chem. Soc.* **126**, 273–279 (2004).

5 Lattuada, M. & Hatton, T. A. Functionalization of monodisperse magnetic nanoparticles. *Langmuir* **23**, 2158–2168 (2007).

6 Stöber, W., Fink, A. & Bohn, E. Controlled growth of monodisperse silica spheres in the micron size range. *J. Colloid Interface Sci.* **26**, 62–69 (1968).

7 Ow, H. *et al.* Bright and stable core–shell fluorescent silica nanoparticles. *Nano Lett.* **5**, 113–117 (2005).

8 Schindelin, J. *et al.* Fiji: an open-source platform for biological-image analysis. *Nat. Methods* **9**, 676–682 (2012).

9 Haiss, W., Thanh, N. T. K., Aveyard, J. & Fernig, D. G. Determination of size and concentration of gold nanoparticles from uv–vis spectra. *Anal. Chem.* **79**, 4215–4221 (2007).

10 Chevallet, M., Luche, S. & Rabilloud, T. Silver staining of proteins in polyacrylamide gels. *Nat. Protocols* **1**, 1852–1858 (2006).

11 Hinderliter, P. M. *et al.* ISDD: A computational model of particle sedimentation, diffusion and target cell dosimetry for in vitro toxicity studies. *Part. Fibre Toxicol.* **7**, 36 (2010).
